# MapReduce Based Parallel Neural Networks in Enabling Large Scale Machine Learning

**DOI:** 10.1155/2015/297672

**Published:** 2015-11-22

**Authors:** Yang Liu, Jie Yang, Yuan Huang, Lixiong Xu, Siguang Li, Man Qi

**Affiliations:** ^1^School of Electrical Engineering and Information, Sichuan University, Chengdu 610065, China; ^2^The Key Laboratory of Embedded Systems and Service Computing, Tongji University, Shanghai 200092, China; ^3^Department of Computing, Canterbury Christ Church University, Canterbury, Kent CT1 1QU, UK

## Abstract

Artificial neural networks (ANNs) have been widely used in pattern recognition and classification applications. However, ANNs are notably slow in computation especially when the size of data is large. Nowadays, big data has received a momentum from both industry and academia. To fulfill the potentials of ANNs for big data applications, the computation process must be speeded up. For this purpose, this paper parallelizes neural networks based on MapReduce, which has become a major computing model to facilitate data intensive applications. Three data intensive scenarios are considered in the parallelization process in terms of the volume of classification data, the size of the training data, and the number of neurons in the neural network. The performance of the parallelized neural networks is evaluated in an experimental MapReduce computer cluster from the aspects of accuracy in classification and efficiency in computation.

## 1. Introduction

Recently, big data has received a momentum from both industry and academia. Many organizations are continuously collecting massive amounts of datasets from various sources such as the World Wide Web, sensor networks, and social networks. In [1], big data is defined as a term that encompasses the use of techniques to capture, process, analyze, and visualize potentially large datasets in a reasonable time frame not accessible to standard IT technologies. Basically, big data is characterized with three Vs [2]:

- Volume: the sheer amount of data generated.
- Velocity: the rate at which the data is being generated.
- Variety: the heterogeneity of data sources.

Artificial neural networks (ANNs) have been widely used in pattern recognition and classification applications. Back-propagation neural network (BPNN), the most popular one of ANNs, could approximate any continuous nonlinear functions by arbitrary precision with an enough number of neurons [3]. Normally, BPNN employs the back-propagation algorithm for training which requires a significant amount of time when the size of the training data is large [4]. To fulfill the potentials of neural networks in big data applications, the computation process must be speeded up with parallel computing techniques such as the Message Passing Interface (MPI) [5, 6]. In [7], Long and Gupta presented a scalable parallel artificial neural network using MPI for parallelization. It is worth noting that MPI was designed for data intensive applications with high performance requirements. MPI provides little support in fault tolerance. If any fault happens, an MPI computation has to be started from the beginning. As a result, MPI is not suitable for big data applications, which would normally run for many hours during which some faults might happen.

This paper presents a MapReduce based parallel back-propagation neural network (MRBPNN). MapReduce has become a de facto standard computing model in support of big data applications [8, 9]. MapReduce provides a reliable, fault-tolerant, scalable, and resilient computing framework for storing and processing massive datasets. MapReduce scales well with ever increasing sizes of datasets due to its use of hash keys for data processing and the strategy of moving computation to the closest data nodes. In MapReduce, there are mainly two functions which are the Map function (mapper) and the Reduce function (reducer). Basically, a mapper is responsible for actual data processing and generates intermediate results in the form of 〈key, value〉 pairs. A reducer collects the output results from multiple mappers with secondary processing including sorting and merging the intermediate results based on the key values. Finally the Reduce function generates the computation results.

We present three MRBPNNs (i.e., MRBPNN_1, MRBPNN_2, and MRBPNN_3) to deal with different data intensive scenarios. MRBPNN_1 deals with a scenario in which the dataset to be classified is large. The input dataset is segmented into a number of data chunks which are processed by mappers in parallel. In this scenario, each mapper builds the same BPNN classifier using the same set of training data. MRBPNN_2 focuses on a scenario in which the volume of the training data is large. In this case, the training data is segmented into data chunks which are processed by mappers in parallel. Each mapper still builds the same BPNN but uses only a portion of the training dataset to train the BPNN. To maintain a high accuracy in classification, we employ a bagging based ensemble technique [10] in MRBPNN_2. MRBPNN_3 targets a scenario in which the number of neurons in a BPNN is large. In this case, MRBPNN_3 fully parallelizes and distributes the BPNN among the mappers in such a way that each mapper employs a portion of the neurons for training.

The rest of the paper is organized as follows.  Section 2 gives a review on the related work.  Section 3 presents the designs and implementations of the three parallel BPNNs using the MapReduce model.  Section 4 evaluates the performance of the parallel BPNNs and analyzes the experimental results.  Section 5 concludes the paper.

## 2. Related Work

ANNs have been widely applied in various pattern recognition and classification applications. For example, Jiang et al. [11] employed a back-propagation neural network to classify high resolution remote sensing images to recognize roads and roofs in the images. Khoa et al. [12] proposed a method to forecast the stock price using BPNN.

Traditionally, ANNs are employed to deal with a small volume of data. With the emergence of big data, ANNs have become computationally intensive for data intensive applications which limits their wide applications. Rizwan et al. [13] employed a neural network on global solar energy estimation. They considered the research as a big task, as traditional approaches are based on extreme simplicity of the parameterizations. A neural network was designed which contains a large number of neurons and layers for complex function approximation and data processing. The authors reported that in this case the training time will be severely affected. Wang et al. [14] pointed out that currently large scale neural networks are one of the mainstream tools for big data analytics. The challenge in processing big data with large scale neural networks includes two phases which are the training phase and the operation phase. To speed up the computations of neural networks, there are some efforts that try to improve the selection of initial weights [15] or control the learning parameters [16] of neural networks. Recently, researchers have started utilizing parallel and distributed computing technologies such as cloud computing to solve the computation bottleneck of a large neural network [17–19]. Yuan and Yu [20] employed cloud computing mainly for exchange of privacy data in a BPNN implementation in processing ciphered text classification tasks. However, cloud computing as a computing paradigm simply offers infrastructure as a service (IaaS), platform as a service (PaaS), and software as a service (SaaS). It is worth noting that cloud computing still needs big data processing models such as the MapReduce model to deal with data intensive applications. Gu et al. [4] presented a parallel neural network using in-memory data processing techniques to speed up the computation of the neural network but without considering the accuracy aspect of the implemented parallel neural network. In this work, the training data is simply segmented into data chunks which are processed in parallel. Liu et al. [21] presented a MapReduce based parallel BPNN in processing a large set of mobile data. This work further employs AdaBoosting to accommodate the loss of accuracy of the parallelized neural network. However, the computationally intensive issue may exist not only at the training phase but also at the classification phase. In addition, AdaBoosting is a popular sampling technique; it may enlarge the weights of wrongly classified instances which would deteriorate the algorithm accuracy.

## 3. Parallelizing Neural Networks

This section presents the design details of the parallelized MRBPNN_1, MRBPNN_2, and MRBPNN_3. First, a brief review of BPNN is introduced.

### 3.1. Back-Propagation Neural Network

**Figure 1:**
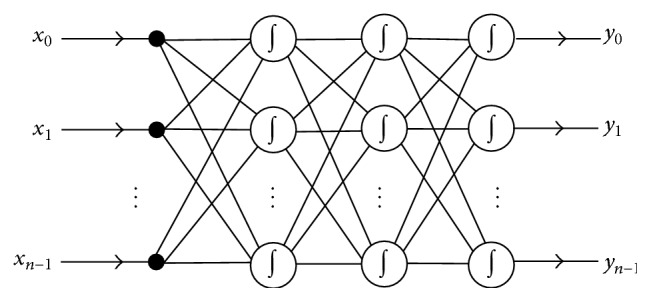
The structure of a typical BPNN.

Back-propagation neural network is a multilayer feed forward network which trains the training data using an error back-propagation mechanism. It has become one of the most widely used neural networks. BPNN can perform a large volume of input-output mappings without knowing their exact mathematical equations. This benefits from the gradient-descent feature of its back-propagation mechanism. During the error propagation, BPNN keeps tuning the parameters of the network until it adapts to all input instances. A typical BPNN is shown in Figure 1, which consists of an arbitrary number of inputs and outputs.

Generally speaking, a BPNN can have multiple network layers. However, it has been widely accepted that a three-layer BPNN would be enough to fit the mathematical equations which approximate the mapping relationships between inputs and outputs [3]. Therefore, the topology of a BPNN usually contains three layers: input layer, one hidden layer, and output layer. The number of inputs in the input layer is mainly determined by the number of elements in an input eigenvector; for instance, let **s** denote an input instance:

(1) $$\begin{array}{c} s=\left\{\;a_{1},a_{2},a_{3},\ldots ,a_{n}\;\right\}. \end{array}$$

Then, the number of inputs is *n*. Similarly, the number of neurons in the output layer is determined by the number of classifications. And the number of neurons in the hidden layer is determined by users. Every input of a neuron has a weight *w*_*ij*_, where *i* and *j* represent the source and destination of the input. Each neuron also maintains an optional parameter *θ*_*j*_ which is actually a bias for varying the activity of the *j*th neuron in a layer. Therefore, let *o*_*j*′_ denote the output from a previous neuron and let  *o*_*j*_ denote the output of this layer; the input *I*_*j*_ of neurons located in both the hidden and output layer can be represented by

(2) $$\begin{array}{c} I_{j}=\underset{i}{\sum }w_{ij}o_{j^{\prime }}+\theta_{j}. \end{array}$$

The output of a neuron is usually computed by the sigmoid function, so the output *o*_*j*_ can be computed by

(3) $$\begin{array}{c} o_{j}=\frac{1}{1+e^{-I_{j}}}. \end{array}$$

After the feed forward process is completed, the back-propagation process starts. Let Err_*j*_ represent the error-sensitivity and let  *t*_*j*_ represent the desirable output of neuron *j* in the output layer; thus,

(4) $$\begin{array}{c} {\text{Err}}_{j}=o_{j}\left(\;1-o_{j}\;\right)\left(\;t_{j}-o_{j}\;\right). \end{array}$$

Let Err_*k*_ represent the error-sensitivity of one neuron in the last layer and let  *w*_*kj*_ represent its weight; thus, Err_*j*_ of a neuron in the other layers can be computed using

(5) $$\begin{array}{c} {\text{Err}}_{j}=o_{j}\left(\;1-o_{j}\;\right)\underset{k}{\sum }{\text{Err}}_{k}w_{kj}. \end{array}$$

After Err_*j*_ is computed, the weights and biases of each neuron are tuned in back-propagation process using

(6) Δwij=Errjoj,wij=wij+Δwij,Δθj=Errj,θj=θj+Δθj.

After the first input vector finishes tuning the network, the next round starts for the following input vectors. The input keeps training the network until (7) is satisfied for a single output or (8) is satisfied for multiple outputs:

(7) min⁡Ee2=min⁡Et−o2,

(8) min⁡EeTe=min⁡Et−oTt−o.⁡

### 3.2. MapReduce Computing Model

MapReduce has become the de facto standard computing model in dealing with data intensive applications using a cluster of commodity computers. Popular implementations of the MapReduce computing model include Mars [22], Phoenix [23], and Hadoop framework [24, 25]. The Hadoop framework has been widely taken up by the community due to its open source feature. Hadoop has its Hadoop Distributed File System (HDFS) for data management. A Hadoop cluster has one name node (Namenode) and a number of data nodes (Datanodes) for running jobs. The name node manages the metadata of the cluster whilst a data node is the actual processing node. The Map functions (mappers) and Reduce functions (reducers) run on the data nodes. When a job is submitted to a Hadoop cluster, the input data is divided into small chunks of an equal size and saved in the HDFS. In terms of data integrity, each data chunk can have one or more replicas according to the cluster configuration. In a Hadoop cluster, mappers copy and read data from either remote or local nodes based on data locality. The final output results will be sorted, merged, and generated by reducers in HDFS.

### 3.3. The Design of MRBPNN_1

MRBPNN_1 targets the scenario in which BPNN has a large volume of testing data to be classified. Consider a testing instance *s*_*i*_ = {*a*_1_, *a*_2_, *a*_3_,…, *a*_*in*_}, *s*_*i*_ ∈ *S*, where

(i) *s*_*i*_ denotes an instance;
(ii) *S* denotes a dataset;
(iii) *in* denotes the length of *s*_*i*_; it also determines the number of inputs of a neural network;
(iv) the inputs are capsulated by a format of 〈instance_*k*_, target_*k*_, type〉;
(v) instance_*k*_ represents *s*_*i*_, which is the input of a neural network;
(vi) target_*k*_ represents the desirable output if instance_*k*_ is a training instance;
(vii) type field has two values, “train” and “test,” which marks the type of instance_*k*_; if “test” value is set, target_*k*_ field should be left empty.

Files which contain instances are saved into HDFS initially. Each file contains all the training instances and a portion of the testing instances. Therefore, the file number *n* determines the number of mappers to be used. The file content is the input of MRBPNN_1.

When the algorithm starts, each mapper initializes a neural network. As a result, there will be *n* neural networks in the cluster. Moreover, all the neural networks have exactly the same structure and parameters. Each mapper reads data in the form of 〈instance_*k*_, target_*k*_, type〉 from a file and parses the data records. If the value of type field is “train,” instance_*k*_ is input into the input layer of the neural network. The network computes the output of each layer using (2) and (3), until the output layer generates an output which indicates the completion of the feed forward process. And then the neural network in each mapper starts the back-propagation process. It computes and updates new weights and biases for its neurons using (4) to (6). The neural network inputs instance_*k*+1_. Repeat the feed forward and back-propagation process until all the instances which are labeled as “train” are processed and the error is satisfied.

Each mapper starts classifying instances labeled as “test” by running the feed forward process. As each mapper only classifies a portion of the entire testing dataset, the efficiency is improved. At last, each mapper outputs intermediate output in the form of 〈instance_*k*_, *o*_*jm*_〉, where instance_*k*_ is the key and *o*_*jm*_ represents the output of the *m*th mapper.

**Figure 2:**
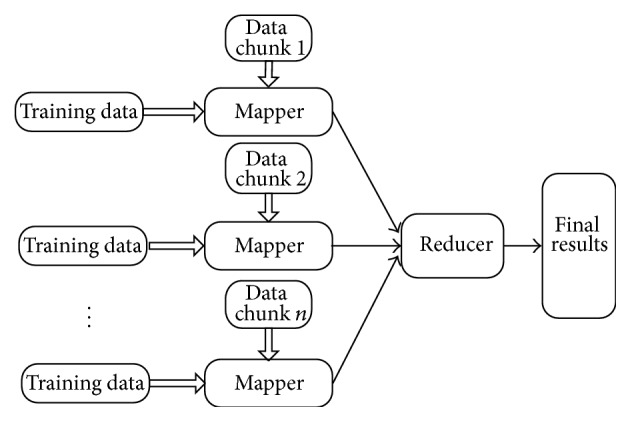
MRBPNN_1 architecture.

One reducer starts collecting and merging all the outputs of the mappers. Finally, the reducer outputs 〈instance_*k*_, *o*_*jm*_〉 into HDFS. In this case, *o*_*jm*_ represents the final classification result of instance_*k*_.  Figure 2 shows the architecture of MRBPNN_1 and Algorithm 1 shows the pseudocode.

### 3.4. The Design of MRBPNN_2

**Figure 3:**
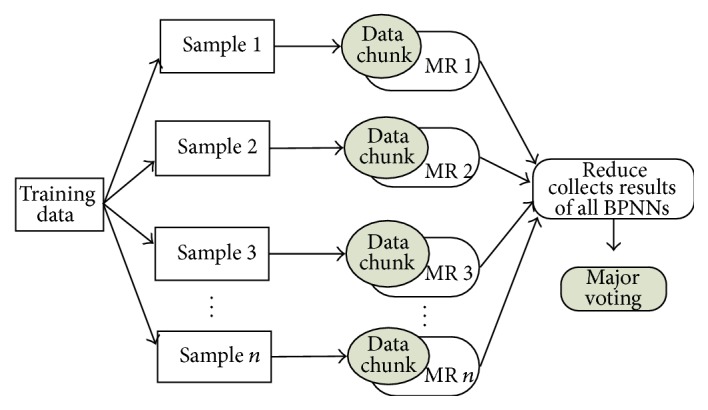
MRBPNN_2 architecture.

MRBPNN_2 focuses on the scenario in which a BPNN has a large volume of training data. Consider a training dataset *S* with a number of instances. As shown in Figure 3, MRBPNN_2 divides *S* into *n* data chunks of which each data chunk *s*_*i*_ is processed by a mapper for training, respectively:

(9) $$\begin{array}{c} S=\overset{n}{\underset{1}{⋃}}{\;s}_{i},\;\left\{\forall s\in s_{i}\;|\;s\notin s_{n},\;\;i\neq n\right\}. \end{array}$$

Each mapper in the Hadoop cluster maintains a BPNN, and each *s*_*i*_ is considered as the input training data for the neural network maintained in mapper_*i*_. As a result, each BPNN in a mapper produces a classifier based on the trained parameters:

(10) $$\begin{array}{c} \left(\;{\text{mapper}}_{i},{{\text{BPNN}}_{i},s}_{i}\;\right)⟶{\text{classifier}}_{i}. \end{array}$$

To reduce the computation overhead, each classifier_*i*_ is trained with a part of the original training dataset. However, a critical issue is that the classification accuracy of a mapper will be significantly degraded using only a portion of the training data. To solve this issue, MRBPNN_2 employs ensemble technique to maintain the classification accuracy by combining a number of weak learners to create a strong learner.

#### 3.4.1. Bootstrapping

Training diverse classifiers from a single training dataset has been proven to be simple compared with the case of finding a strong learner [26]. A number of techniques exist for this purpose. A widely used technique is to resample the training dataset based on bootstrap aggregating such as bootstrapping and majority voting. This can reduce the variance of misclassification errors and hence increases the accuracy of the classifications.

As mentioned in [26], balanced bootstrapping can reduce the variance when combining classifiers. Balanced bootstrapping ensures that each training instance equally appears in the bootstrap samples. It might not be always the case that each bootstrapping sample contains all the training instances. The most efficient way of creating balanced bootstrap samples is to construct a string of instances *X*_1_, *X*_2_, *X*_3_,…, *X*_*n*_ repeating *B* times so that a sequence of *Y*_1_, *Y*_2_, *Y*_3_,…, *Y*_*Bn*_ can be achieved. A random permutation *p* of the integers from 1 to *B*_*n*_ is taken. Therefore, the first bootstrapping sample can be created from *Y*_*p*_(1), *Y*_*p*_(2), *Y*_*p*_(3),…, *Y*_*p*_(*n*). In addition, the second bootstrapping sample is created from *Y*_*p*_(*n* + 1), *Y*_*p*_(*n* + 2), *Y*_*p*_(*n* + 3),…, *Y*_*p*_(2*n*) and the process continues until *Y*_*p*_((*B* − 1)*n* + 1), *Y*_*p*_((*B* − 1)*n* + 2), *Y*_*p*_((*B* − 1)*n* + 3),…, *Y*_*p*_(*Bn*) is the *B*th bootstrapping sample. The bootstrapping samples can be used in bagging to increase the accuracy of classification.

#### 3.4.2. Majority Voting

This type of ensemble classifiers performs classifications based on the majority votes of the base classifiers [26]. Let us define the prediction of the *i*th classifier *P*_*i*_ as *P*_*i*,*j*_ ∈ {1,0}, *i* = 1,…, *I* and *j* = 1,…, *c*, where *I* is the number of classifiers and *c* is the number of classes. If the *i*th classifier chooses class *j*, then *P*_*i*,*j*_ = 1; otherwise, *P*_*i*,*j*_ = 0. Then, the ensemble prediction for class *k* is computed using

(11) $$\begin{array}{c} P_{i,k}=\max_{j=1}^{c}\overset{I}{\underset{i=1}{\sum }}P_{i,j}. \end{array}$$

#### 3.4.3. Algorithm Design

At the beginning, MRBPNN_2 employs balanced bootstrapping to generate a number of subsets of the entire training dataset:

(12) balanced  bootstrapping⟶S1,S2,S3,…,Sn,⋃i=1n Si=S,

where *S*_*i*_ represents the *i*th subset, which belongs to entire dataset *S*. *n* represents the total number of subsets.

Each *S*_*i*_ is saved in one file in HDFS. Each instance *s*_*k*_ = {*a*_1_, *a*_2_, *a*_3_,…, *a*_*in*_}, *s*_*k*_ ∈ *S*_*i*_, is defined in the format of 〈instance_*k*_, target_*k*_, type〉, where

(i) instance_*k*_ represents one bootstrapped instance *s*_*k*_, which is the input of neural network;
(ii) *in* represents the number of inputs of the neural network;
(iii) target_*k*_ represents the desirable output if instance_*k*_ is a training instance;
(iv) type field has two values, “train” and “test,” which marks the type of instance_*k*_; if “test” value is set, target_*k*_ field should be left empty.

When MRBPNN_2 starts, each mapper constructs one BPNN and initializes weights and biases with random values between −1 and 1 for its neurons. And then a mapper inputs one record in the form of 〈instance_*k*_, target_*k*_, type〉 from the input file.

The mapper firstly parses the data and retrieves the type of the instance. If the type value is “train,” the instance is fed into the input layer. Secondly, each neuron in different layers computes its output using (2) and (3) until the output layer generates an output which indicates the completion of the feed forward process. Each mapper starts a back-propagation process and computes and updates weights and biases for neurons using (4) to (6). The training process finishes until all the instances marked as “train” are processed and error is satisfied. All the mappers start feed forwarding to classify the testing dataset. In this case, each neural network in a mapper generates the classification result of an instance at the output layer. Each mapper generates an intermediate output in the form of 〈instance_*k*_, *o*_*jm*_〉, where instance_*k*_ is the key and *o*_*jm*_ represents the outputs of the *m*th mapper.

Finally, a reducer collects the outputs of all the mappers. The outputs with the same key are merged together. The reducer runs majority voting using (11) and outputs the result of instance_*k*_ into HDFS in the form of 〈instance_*k*_, *r*_*k*_〉, where *r*_*k*_ represents the voted classification result of instance_*k*_. Figure 3 shows the algorithm architecture and Algorithm 2 presents the pseudocode of MRBPNN_2.

### 3.5. The Design of MRBPNN_3

MRBPNN_3 aims at the scenario in which a BPNN has a large number of neurons. The algorithm enables an entire MapReduce cluster to maintain one neural network across it. Therefore, each mapper holds one or several neurons.

There are a number of iterations that exist in the algorithm with *l* layers. MRBPNN_3 employs a number of *l* − 1  MapReduce jobs to implement the iterations. The feed forward process runs in *l* − 1 rounds whilst the back-propagation process occurs only in the last round. A data format in the form of 〈index_*k*_, instance_*n*_, *w*_*ij*_, *θ*_*j*_, target_*n*_, {*w*_*ij*_^2^, *θ*_*j*_^2^,…, *w*_*ij*_^*l*−1^, *θ*_*j*_^*l*−1^}〉 has been designed to guarantee the data passing between Map and Reduce operations, where

(i) index_*k*_ represents the *k*th reducer;
(ii) instance_*n*_ represents the *n*th training or testing instance of the dataset; one instance is in the form of instance_*n*_ = {*a*_1_, *a*_2_,…, *a*_*in*_}, where *in* is length of the instance;
(iii) *w*_*ij*_ represents a set of weights of an input layer, whilst *θ*_*j*_ represents the biases of the neurons in the first hidden layer;
(iv) target_*n*_ represents the encoded desirable output of a training instance instance_*n*_;
(v) the list of {*w*_*ij*_^2^, *θ*_*j*_^2^,…, *w*_*ij*_^*l*−1^, *θ*_*j*_^*l*−1^} represents the weights and biases for next layers; it can be extended based on the layers of the network; for a standard three-layer neural network, this option becomes {*w*_*ijo*_, *θ*_*jo*_}.

Before MRBPNN_3 starts, each instance and the information defined by data format are saved in one file in HDFS. The number of the layers is determined by the length of {*w*_*ij*_^2^, *θ*_*j*_^2^ …, *w*_*ij*_^*l*−1^, *θ*_*j*_^*l*−1^} field. The number of neurons in the next layer is determined by the number of files in the input folder. Generally, different from MRBPNN_1 and MRBPNN_2, MRBPNN_3 does not initialize an explicit neural network; instead, it maintains the network parameters based on the data defined in the data format.

When MRBPNN_3 starts, each mapper initially inputs one record from HDFS. And then it computes the output of a neuron using (2) and (3). The output is generated by a mapper, which labels index_*k*_ as a key and the neuron's output as a value in the form of

  〈index_*k*_, *o*_*j*_, {*w*_*ij*_^2^, *θ*_*j*_^2^ …, *w*_*ij*_^*l*−1^, *θ*_*j*_^*l*−1^}, target_*n*_〉, where *o*_*j*_ represents the neuron's output.

Parameter index_*k*_ can guarantee that the *k*th reducer collects the output, which maintains the neural network structure. It should be mentioned that if the record is the first one processed by MRBPNN_3, {*w*_*ij*_^2^, *θ*_*j*_^2^ …, *w*_*ij*_^*l*−1^, *θ*_*j*_^*l*−1^} will be also initialized with random values between −1 and 1 by the mappers. The *k*th reducer collects the results from the mappers in the form of 〈index_*k*′_, *o*_*j*_, {*w*_*ij*_^2^, *θ*_*j*_^2^ …, *w*_*ij*_^*l*−1^, *θ*_*j*_^*l*−1^}, target_*n*_〉. These *k* reducers generate *k* outputs. The index_*k*′_ of the reducer output explicitly tells the *k*′th mapper to start processing this output file. Therefore, the number of neurons in the next layer can be determined by the number of reducer output files, which are the input data for the next layer neurons. Subsequently, mappers start processing their corresponding inputs by computing (2) and (3) using *w*_*ij*_^2^ and *θ*_*j*_^2^.

**Figure 4:**
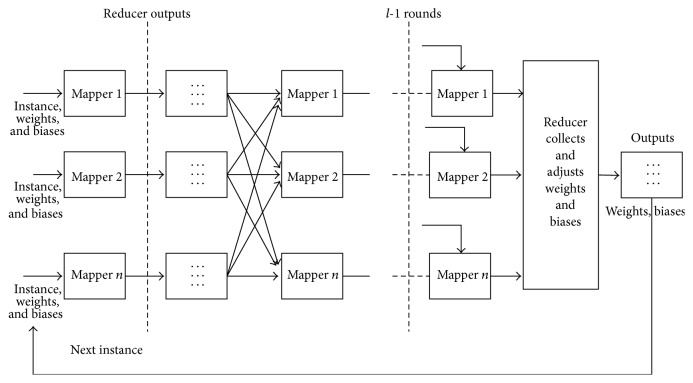
MRBPNN_3 structure.

The above steps keep looping until reaching the last round. The processing of this last round consists of two steps. The first step is that mappers also process 〈index_*k*′_, *o*_*j*_, {*w*_*ij*_^*l*−1^, *θ*_*j*_^*l*−1^}, target_*n*_〉, compute neurons' outputs, and generate results in the forms of 〈*o*_*j*_, target_*n*_〉. One reducer collects the output results of all the mappers in the form of 〈*o*_*j*1_, *o*_*j*2_, *o*_*j*3_,…, *o*_*jk*_, target_*n*_〉. In the second step, the reducer executes the back-propagation process. The reducer computes new weights and biases for each layer using (4) to (6). MRBPNN_3 retrieves the previous outputs, weights, and biases from the input files of mappers, and then it writes the updated weights and biases *w*_*ij*_, *θ*_*j*_, {*w*_*ij*_^2^, *θ*_*j*_^2^,…, *w*_*ij*_^*l*−1^, *θ*_*j*_^*l*−1^} into the initial input file in the form of 〈index_*k*_, instance_*n*_, *w*_*ij*_, *θ*_*j*_, target_*n*_, {*w*_*ij*_^2^, *θ*_*j*_^2^,…, *w*_*ij*_^*l*−1^, *θ*_*j*_^*l*−1^}〉. The reducer reads the second instance in the form of 〈instance_*n*+1_, target_*n*+1_〉 for which the fields instance_*n*_ and target_*n*_ in the input file are replaced by instance_*n*+1_ and target_*n*+1_. The training process continues until all the instances are processed and error is satisfied. For classification, MRBPNN_3 only needs to run the feed forwarding process and collects the reducer output in the form of 〈*o*_*j*_, target_*n*_〉.  Figure 4 shows three-layer architecture of MRBPNN_3 and Algorithm 3 presents the pseudocode.

## 4. Performance Evaluation

We have implemented the three parallel BPNNs using Hadoop, an open source implementation framework of the MapReduce computing model. An experimental Hadoop cluster was built to evaluate the performance of the algorithms. The cluster consisted of 5 computers in which 4 nodes are Datanodes and the remaining one is Namenode. The cluster details are listed in Table 1.

Two testing datasets were prepared for evaluations. The first dataset is a synthetic dataset. The second is the Iris dataset which is a published machine learning benchmark dataset [27].  Table 2 shows the details of the two datasets.

We implemented a three-layer neural network with 16 neurons in the hidden layer. The Hadoop cluster was configured with 16 mappers and 16 reducers. The number of instances was varied from 10 to 1000 for evaluating the precision of the algorithms. The size of the datasets was varied from 1 MB to 1 GB for evaluating the computation efficiency of the algorithms. Each experiment was executed five times and the final result was an average. The precision *p* is computed using

(13) $$\begin{array}{c} p=\frac{r}{r+w}\times 100\%, \end{array}$$

where *r* represents the number of correctly recognized instances. *w* represents the number of wrongly recognized instances. *p* represents the precision.

### 4.1. Classification Precision

**Figure 5:**
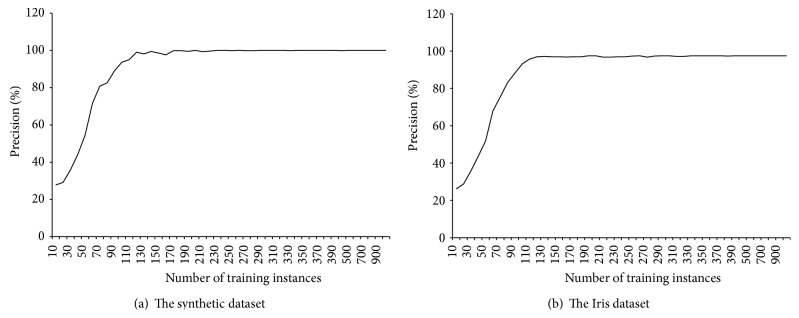
The precision of MRBPNN_1 on the two datasets.

The classification precision of MRBPNN_1 was evaluated using a varied number of training instances. The maximum number of the training instances was 1000 whilst the maximum number of the testing instances was also 1000. The large number of instances is based on the data duplication.  Figure 5 shows the precision results of MRBPNN_1 in classification using 10 mappers. It can be observed that the precision keeps increasing with an increase in the number of training instances. Finally, the precision of MRBPNN_1 on the synthetic dataset reaches 100% while the precision on the Iris dataset reaches 97.5%. In this test, the behavior of the parallel MRBPNN_1 is quite similar to that of the standalone BPNN. The reason is that MRBPNN_1 does not distribute the BPNN among the Hadoop nodes; instead, it runs on Hadoop to distribute the data.

**Figure 6:**
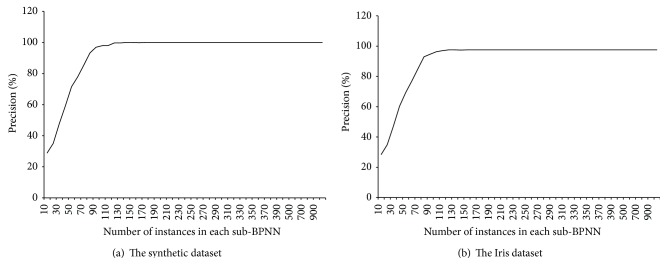
The precision of MRBPNN_2 on the two datasets.

To evaluate MRBPNN_2, we designed 1000 training instances and 1000 testing instances using data duplication. The mappers were trained by subsets of the training instances and produced the classification results of 1000 testing instances based on bootstrapping and majority voting. MRBPNN_2 employed 10 mappers each of which inputs a number of training instances varying from 10 to 1000.  Figure 6 presents the precision results of MRBPNN_2 on the two testing datasets. It also shows that, along with the increasing number of training instances in each subneural network, the achieved precision based on majority voting keeps increasing. The precision of MRBPNN_2 on the synthetic dataset reaches 100% whilst the precision on the Iris dataset reaches 97.5%, which is higher than that of MRBPNN_1.

**Figure 7:**
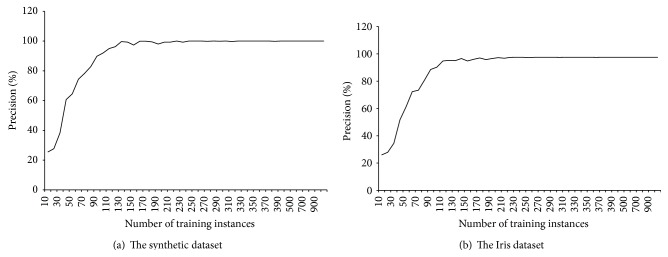
The precision of MRBPNN_3 on the two datasets.

MRBPNN_3 implements a fully parallel and distributed neural network using Hadoop to deal with a complex neural network with a large number of neurons.  Figure 7 shows the performance of MRBPNN_3 using 16 mappers. The precision also increases along with the increasing number of training instances for both datasets. It also can be observed that the stability of the curve is quite similar to that of MRBPNN_1. Both curves have more fluctuations than that of MRBPNN_2.

**Figure 8:**
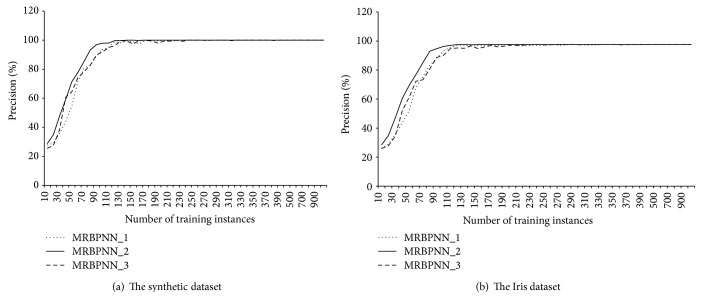
Precision comparison of the three parallel BPNNs.

Figure 8 compares the overall precision of the three parallel BPNNs. MRBPNN_1 and MRBPNN_3 perform similarly, whereas MRBPNN_2 performs the best using bootstrapping and majority voting. In addition, the precision of MRBPNN_2 in classification is more stable than that of both MRBPNN_1 and MRBPNN_3.

**Figure 9:**
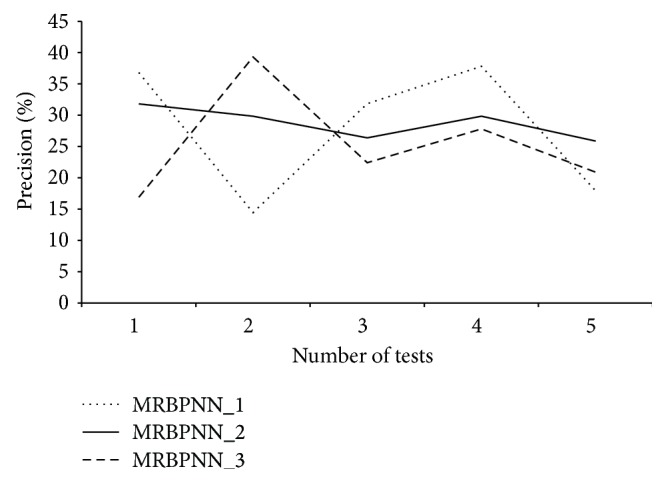
The stability of the three parallel BPNNs.

Figure 9 presents the stability of the three algorithms on the synthetic dataset showing the precision of MRBPNN_2 in classification is highly stable compared with that of both MRBPNN_1 and MRBPNN_3.

### 4.2. Computation Efficiency

**Figure 10:**
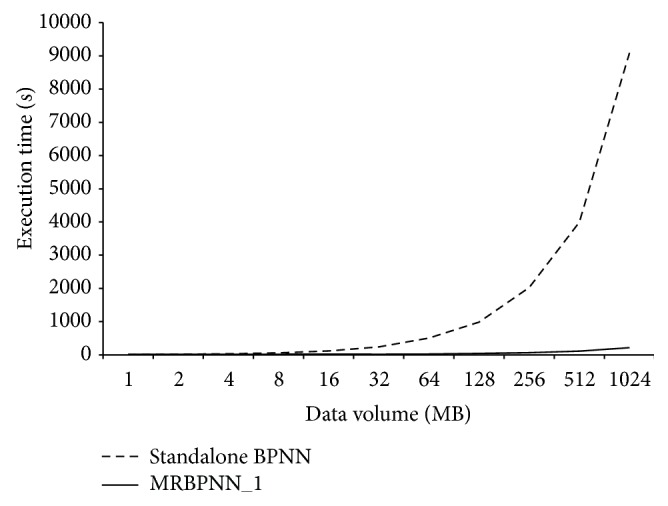
Computation efficiency of MRBPNN_1.

A number of experiments were carried out in terms of computation efficiency using the synthetic dataset. The first experiment was to evaluate the efficiency of MRBPNN_1 using 16 mappers. The volume of data instances was varied from 1 MB to 1 GB.  Figure 10 clearly shows that the parallel MRBPNN_1 significantly outperforms the standalone BPNN. The computation overhead of the standalone BPNN is low when the data size is less than 16 MB. However, the overhead of the standalone BPNN increases sharply with increasing data sizes. This is mainly because MRBPNN_1 distributes the testing data into 4 data nodes in the Hadoop cluster, which runs in parallel in classification.

**Figure 11:**
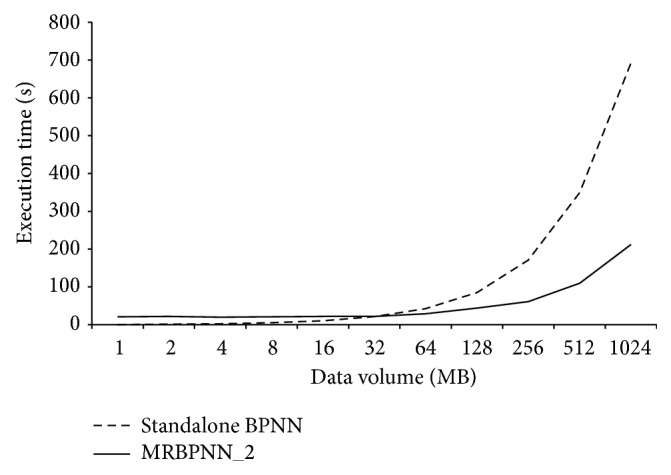
Computation efficiency of MRBPNN_2.

Figure 11 shows the computation efficiency of MRBPNN_2 using 16 mappers. It can be observed that when the data size is small, the standalone BPNN performs better. However, the computation overhead of the standalone BPNN increases rapidly when the data size is larger than 64 MB. Similar to MRBPNN_1, the parallel MRBPNN_2 scales with increasing data sizes using the Hadoop framework.

**Figure 12:**
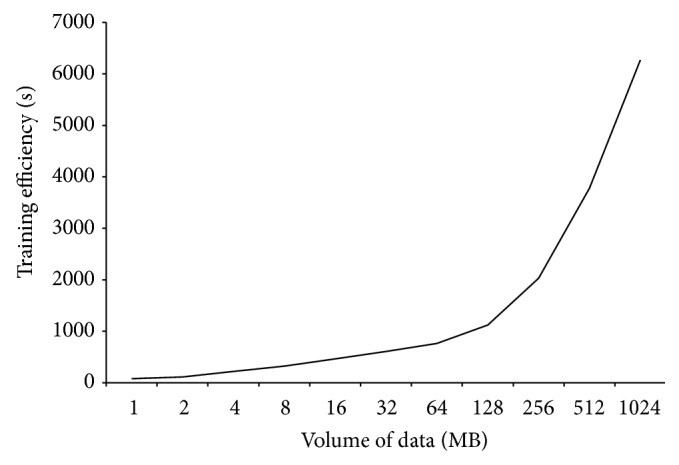
Computation efficiency of MRBPNN_3.

Figure 12 shows the computation overhead of MRBPNN_3 using 16 mappers. MRBPNN_3 incurs a higher overhead than both MRBPNN_1 and MRBPNN_2. The reason is that both MRBPNN_1 and MRBPNN_2 run training and classification within one MapReduce job, which means mappers and reducers only need to start once. However, MRBPNN_3 contains a number of jobs. The algorithm has to start mappers and reducers a number of times. This process incurs a large system overhead which affects its computation efficiency. Nevertheless, Figure 12 shows the feasibility of fully distributing a BPNN in dealing with a complex neural network with a large number of neurons.

## 5. Conclusion

In this paper, we have presented three parallel neural networks (MRBPNN_1, MRBPNN_2, and MRBPNN_3) based on the MapReduce computing model in dealing with data intensive scenarios in terms of the size of classification dataset, the size of the training dataset, and the number of neurons, respectively. Overall, experimental results have shown the computation overhead can be significantly reduced using a number of computers in parallel. MRBPNN_3 shows the feasibility of fully distributing a BPNN in a computer cluster but incurs a high overhead of computation due to continuous starting and stopping of mappers and reducers in Hadoop environment. One of the future works is to research in-memory processing to further enhance the computation efficiency of MapReduce in dealing with data intensive tasks with many iterations.

## Figures and Tables

**Algorithm 1:**
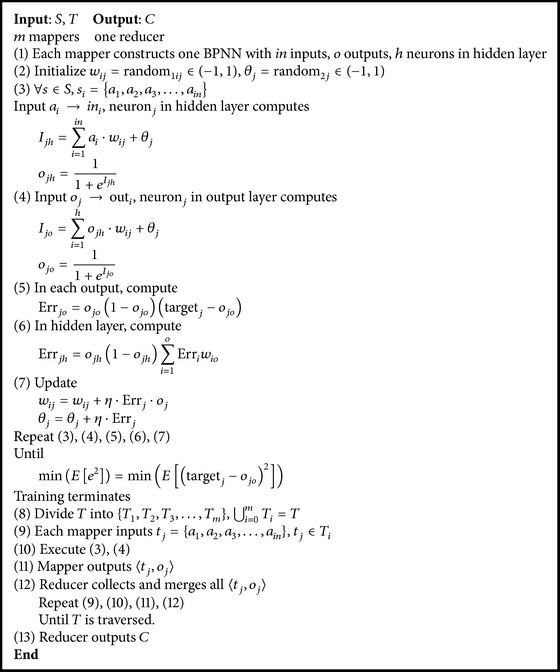
MRBPNN_1.

**Algorithm 2:**
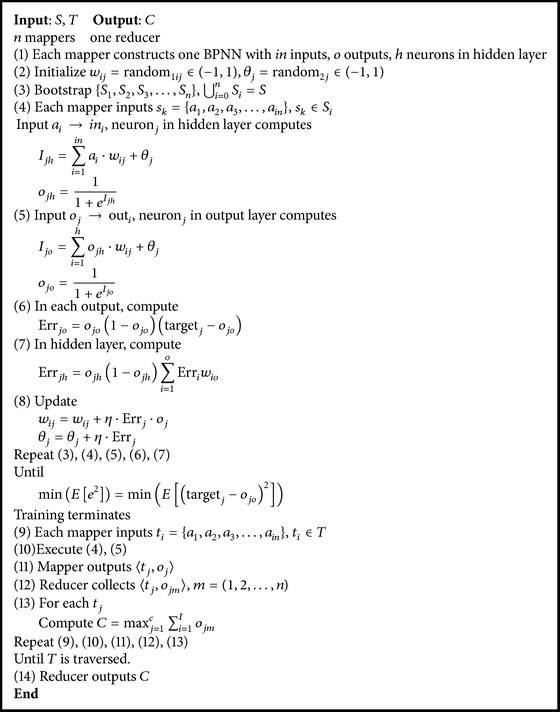
MRBPNN_2.

**Algorithm 3:**
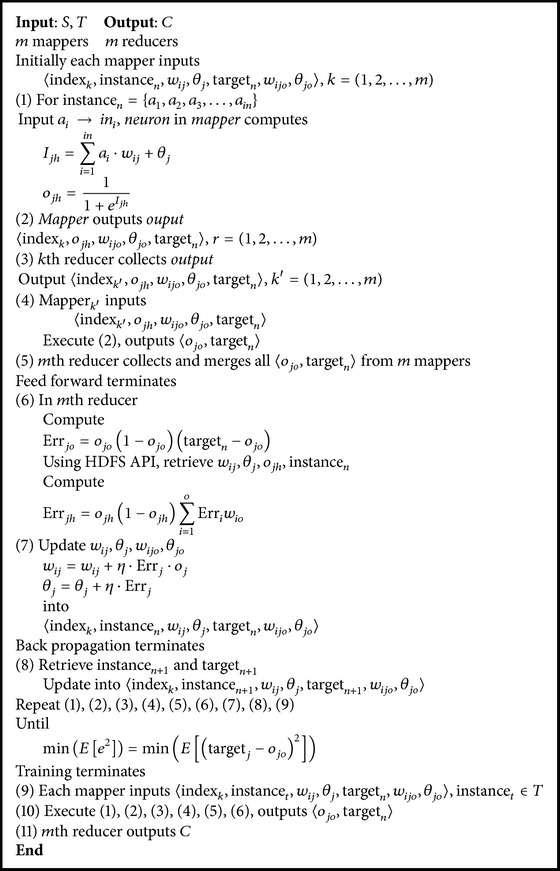
MRBPNN_3.

**Table 1:** Cluster details.

Namenode	CPU: Core i7@3 GHz Memory: 8 GB SSD: 750 GB OS: Fedora

Datanodes	CPU: Core i7@3.8 GHz Memory: 32 GB SSD: 250 GB OS: Fedora

Network bandwidth	1 Gbps

Hadoop version	2.3.0, 32 bits

**Table 2:** Dataset details.

Data type	Instance number	Instance length	Element range	Class number
Synthetic data	200	32	0 and 1	4
Iris data	150	4	(0, 8)	3
